# Low-dose 17α-ethinyl estradiol (EE) exposure exacerbates lupus renal disease and modulates immune responses to TLR7/9 agonists in genetically autoimmune-prone mice

**DOI:** 10.1038/s41598-020-62124-6

**Published:** 2020-03-23

**Authors:** Michael R. Edwards, Rujuan Dai, Bettina Heid, Catharine Cowan, Stephen R. Werre, Thomas Cecere, S. Ansar Ahmed

**Affiliations:** 1 Infectious Disease Research Facility (IDRF), Department of Biomedical Sciences and Pathobiology, VA-MD College of Vet. Medicine, Virginia Tech, Blacksburg, VA USA; 2 Population Health Sciences, VA-MD College of Vet. Medicine, Virginia Tech, Blacksburg, VA USA

**Keywords:** Autoimmunity, Lupus nephritis

## Abstract

Estrogens have been shown to regulate the immune system and modulate multiple autoimmune diseases. 17α-ethinyl estradiol (EE), a synthetic analog of 17β-estradiol, is prescribed commonly and found in oral contraceptives and hormone replacement therapies. Surprisingly, few studies have investigated the immunoregulatory effects of exposure to EE, especially in autoimmunity. In this study, we exposed autoimmune-prone female MRL/*lpr* mice to a human-relevant dose of EE through the oral route of exposure. Since lupus patients are prone to infections, groups of mice were injected with viral (Imiquimod, a TLR7 agonist) or bacterial (ODN 2395, a TLR9 agonist) surrogates. We then evaluated autoimmune disease parameters, kidney disease, and response to *in vivo* TLR7/9 pathogenic signals. EE-exposed mice had increased proteinuria as early as 7 weeks of age. Proteinuria, blood urea nitrogen, and glomerular immune complex deposition were also exacerbated when compared to controls. Production of cytokines by splenic leukocytes were altered in EE-exposed mice. Our study shows that oral exposure to EE, even at a very low dose, can exacerbate azotemia, increase clinical markers of renal disease, enhance glomerular immune complex deposition, and modulate TLR7/9 cytokine production in female MRL/*lpr* mice. This study may have implications for EE-exposure risk for genetically lupus-prone individuals.

## Introduction

Environmental exposure of animals and people to low doses of estrogenic endocrine disrupting chemicals (EEDCs) occurs through a variety of sources, including ingestion of food and water, pharmaceuticals, outdoor activities, pesticides and fertilizers in agricultural applications, industrial chemicals, plastics, sewage, detergents, and cosmetics^1^. One such EEDC, 17α-ethinyl estradiol (EE), a synthetic analog of endogenously produced 17β-estradiol (E2), is a primary component in oral contraceptive pills (OCP) and has been widely used in hormone replacement therapy (HRT). EE is also used as a treatment for breast cancer, vasomotor symptoms in menopause, female hypogonadism, hirsutism, acne vulgaris, and dysmenorrhea^2^.

EE has been found in aquatic environments and water sources even after water treatment has been performed^3^. The high likelihood of chronic or repeated human exposure to multiple EEDCs throughout one’s lifetime is a significant health concern. Although EE is commonly prescribed, the immunomodulatory effects are not yet well understood. It is likely that there may be subsets of populations, such as autoimmune-prone individuals, who may be particularly vulnerable to the effects of EE exposure.

Estrogens have been shown to alter various functions of the immune system and regulate the responses to stimuli in both normal and autoimmune individuals through estrogen receptor-dependent or -independent mechanisms^4–12^. Another such EEDC, bisphenol A (BPA) has been shown to dysregulate the immune system and may promote autoimmunity^13,14^. We recently compared the immunoregulatory effects of pharmaceutical administration of EE and E2 as silastic implants in autoimmune-prone NZB/W_F1_ mice. EE has both common and unique immunoregulatory effects on T cell subsets, cytokine production and gene expression^9,15^. Notably, high dose EE reduced IFNα and IL-6 production from splenic leukocytes following TLR9 stimulation^9^. EE’s recent addition to the European Union’s watch list of chemicals found in surface water, by Directive 2013/39/EU, suggests a broader concern with regards to toxicity and disease development following human exposures^16^.

Kidney failure and infections are two of the primary causes of mortality in lupus patients^17,18^. Autoantibodies bind to autoantigens and form immune-complexes that can accumulate in the glomeruli, resulting in further damage to kidneys and reduced filtration^19^. The reduced renal filtration leads to accumulation of toxins in the blood stream, including blood urea nitrogen (BUN) and creatinine, and glomerular damage allows protein to leak into the urine. Clinically, BUN and proteinuria can be used as biomarkers of kidney disease progression in a living patient.

Infections are common and are one of the leading causes of mortality in lupus patients^17,18^. Viral infections, such as Epstein-Barr virus, have also been associated with SLE development in human patients^20–22^. Toll-like receptor 7 (TLR7) and TLR9, which recognize single-stranded RNA and bacterial DNA, respectively, have been previously associated with lupus disease severity in mice^23–26^. TLR7 knock-out mice had reduced disease severity, while TLR9 knock-out mice had exacerbation of disease parameters^27^. Currently, the specific contributions of TLR7/9 signaling to lupus pathogenesis are unclear. We hypothesized that chronic oral EE exposure at a human-relevant dose would exacerbate autoimmune renal disease and lead to an impaired response to *in vivo* TLR7 and TLR9 challenge. Since SLE patients are more highly susceptible to infections, and we previously showed that EE exposure of autoimmune-prone NZB/W_F1_ mice reduced IFNα following *in vitro* TLR9 stimulation, here, we investigated the effects of chronic low-dose oral EE exposure on autoimmune disease and used TLR7 (Imiquimod) and TLR9 (ODN 2395) agonists as surrogates to mimic viral and bacterial infections *in vivo*. To date, the above aspects have thus far not been investigated.

## Results

### Low-dose EE exposure increases clinical parameters of renal disease

**Figure 1:**
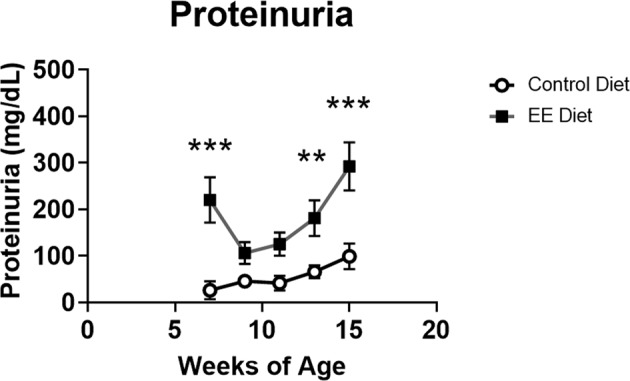
EE exacerbates proteinuria in female autoimmune-prone MRL/lpr mice. Urine protein concentration of all mice was monitored every 2 weeks starting at 7 weeks of age and continued throughout the study. Urine concentration was measured using a dipstick. Multiple time points of the urine protein concentration of mice fed either the control diet or EE diet, and given *in vivo* sterile saline administered *in vivo* are represented. Graph data are expressed as mean ± SEM (n = 9–10 per group). **p < 0.01 ***p < 0.001.

Diet consumption and body weight among all groups were comparable throughout the study (data not shown). Female MRL/lpr mice develop severe immune-complex glomerulonephritis by 16 weeks of age^28–33^. Proteinuria, a measurable indicator of glomerular damage, is typically found to be detectable around 7–9 weeks of age in MRL/lpr female mice, and progressively increases with age. In this study, MRL/lpr mice exposed to low-dose EE diet starting at 3 weeks of age, remarkably, had significantly higher levels of proteinuria as early as four weeks after treatment (7 weeks of age, the first timepoint studied) compared to control-fed mice (Fig. 1). Typically, at this age untreated MRL/lpr mice do not have high levels of proteinuria. Over the entire follow-up period, levels of proteinuria were greater in EE-exposed mice (leastsquares means 184.9, standard error 13.9) compared to control diet fed mice (leastsquares means 55.5, standard error 14.2) (p < 0.001) (Fig. 1).

**Figure 2:**
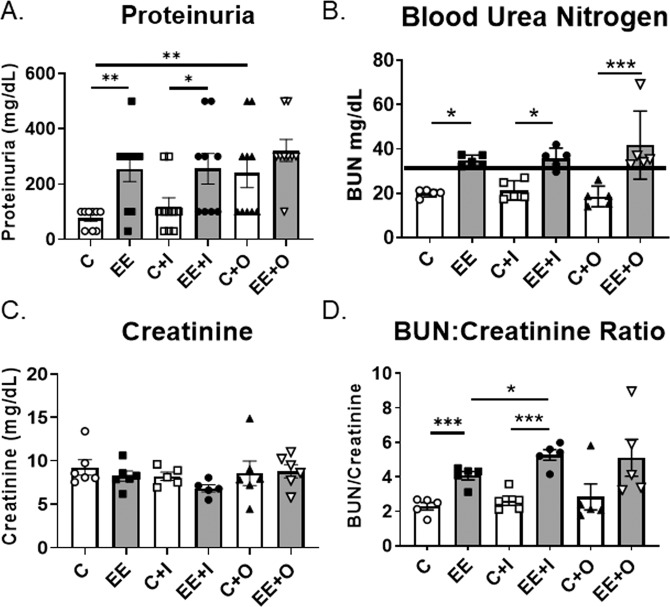
Clinical markers of azotemia and kidney disease are increased by EE exposure. (**A**) Proteinuria was evaluated using the dipstick method. (**B**) Blood urea nitrogen (BUN) was measured in the serum of mice. The high end of normal serum BUN in mice is 33 mg/dL. (**C**) Serum creatinine was analyzed using a commercial colorimetric kit. (**D**) BUN values were divided by creatinine values to determine the BUN:Creatinine ratio. Graph data are expressed as mean ± SEM (n = 5–6 per group). *p < 0.05, **p < 0.01, ***p < 0.001.

It has previously been shown that *in vivo* stimulation of autoimmune prone mice with TLR7 and TLR9 agonists can exacerbate kidney disease^34–36^. We also evaluated the clinical parameters of kidney disease in mice stimulated *in vivo* with imiquimod (a TLR7 agonist as a surrogate for viral exposure) and ODN 2395 (a TLR9 agonist, as a surrogate for bacterial exposure). As indicated, mice fed the EE diet had significantly higher proteinuria than those fed the control diet (Fig. 2A). *In vivo* administration of imiquimod to mice exposed to EE or control diet did not alter the renal disease parameters (Fig. 2A). However, mice fed the control diet and stimulated *in vivo* with ODN 2395 had increased proteinuria compared to control diet mice administered sterile saline (Fig. 2A; C *vs* C + O). Interestingly, proteinuria was significantly elevated with either exposure to EE or stimulation by ODN 2395 (Fig. 2A, C vs EE, C vs C + O). However, the combination of treatments did not result in further elevation of proteinuria, since there was no difference between the EE and EE with ODN 2395 treatment groups (Fig. 2A; EE vs EE + O).

Blood urea nitrogen (BUN) and creatinine are molecules found in the serum that are also measurable indicators of kidney disease progression. No differences in BUN or creatinine were observed among the groups fed the control diet (Fig. 2B,C; C vs C + I vs C + O). Mice exposed to EE had increased serum levels of BUN, regardless of *in vivo* stimulant administered when compared to respective control groups (Fig. 2B, C vs EE, C + I vs EE + I, and C + O vs EE + O). Of note, all mice fed the control diet had serum BUN levels within the normal range, while all mice fed the EE diet were azotemic and had serum BUN levels above the normal threshold for mice (33 mg/dL) (Fig. 2B). No significant differences in serum creatinine were found among groups (Fig. 2C)(post-hoc power = 0.256). The BUN:Creatinine ratio was significantly elevated in mice exposed to EE when compared to the mice fed the control diet (Fig. 2D). Taken together, these data support that mice exposed to EE have azotemia and increased clinical parameters of renal disease compared to the control group. The *in vivo* stimulants administered did not appear to exert a major influence on clinical parameters of renal disease in this study.

### EE exposure increases glomerular immune-complex deposition

**Figure 3:**
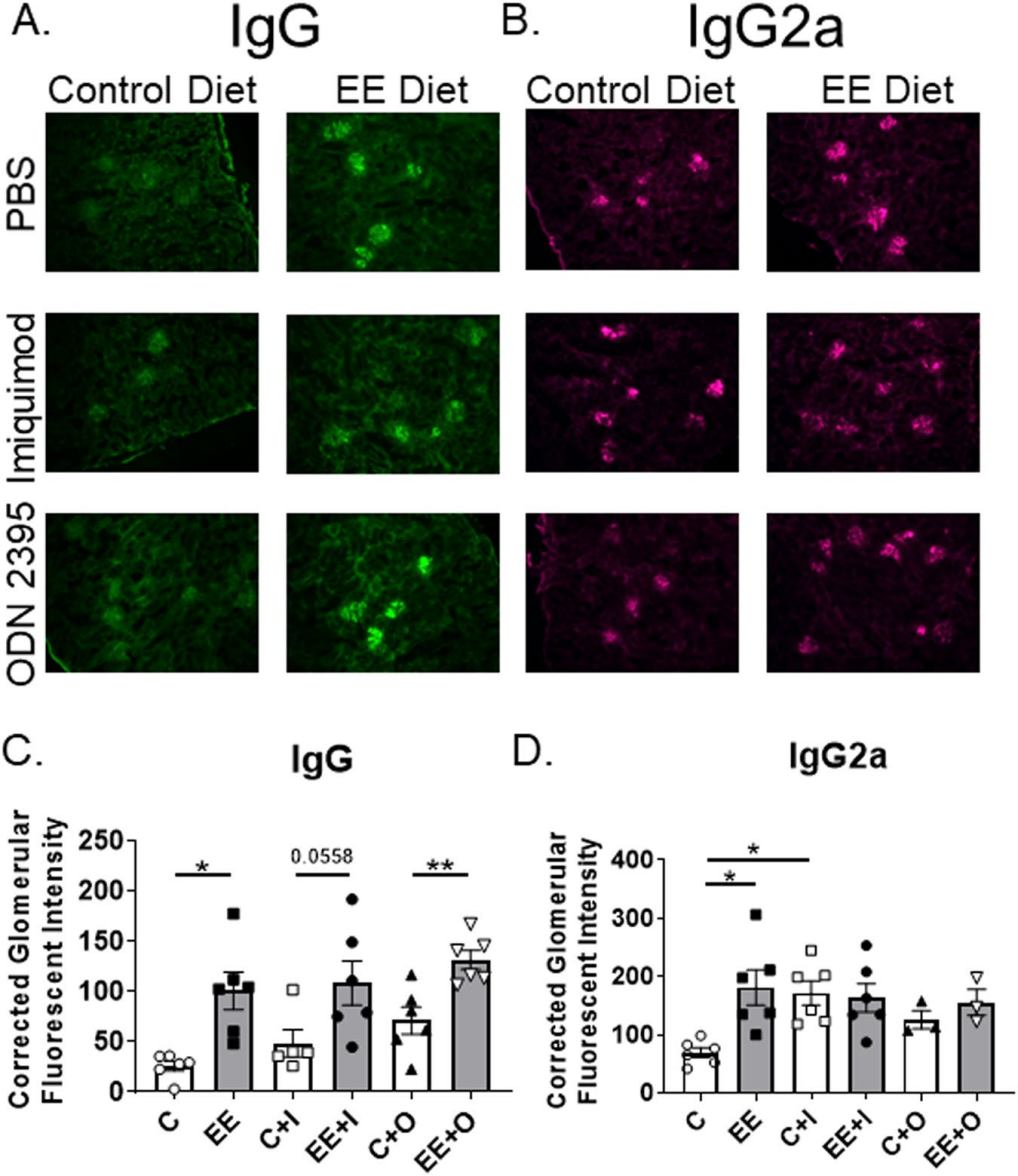
EE exposure augments immune complex deposition (**A**) Representative micrographs of immune-fluorescently labeled IgG and **(B**) IgG2a immune-complex deposition in OCT frozen renal sections (20x magnification). IgG is shown in green, and IgG2a is shown in purple. (**C**,**D**) Corrected glomerular fluorescent intensity of IgG (**C**) and IgG2a (**D**) per glomerulus as measured by Fiji/ImageJ. Twenty glomeruli were analyzed per mouse. Graphical data are expressed as mean ± SEM (n = 6 mice per group). *p < 0.05, **p < 0.01, ***p < 0.001.

Having observed increased clinical parameters of kidney disease in EE-exposed mice, further evaluation of renal structure was warranted. In MRL/lpr mice, akin to human lupus patients, a hallmark of renal disease is deposition of immune complexes in the glomeruli. Circulating autoantibodies can directly target renal podocytes, or become deposited in glomeruli when bound to antigen as immune complexes, promoting renal damage and exacerbation of kidney disease^19^. Total IgG deposition was increased in all groups of mice exposed to EE when compared to the respective control group (Fig. 3A,C; C vs EE, C + I vs EE + I, and C + O vs EE + O). Secondary TLR7 and TLR9 stimulation did not promote IgG deposition in either control or EE diet group. IgG subclass IgG2a deposition was also increased in EE exposed mice with no secondary stimulation (Fig. 3A–D; C vs EE). Interestingly, imiquimod administration only increased IgG2a deposition in mice fed on the control diet (Fig. 3B,D; C vs C + I). Complement C3 was also evaluated, and the measurement of fluorescent intensity was no stronger than the background fluorescence (data not shown). The above data show that even a very low dose of EE in the diet promotes deposition of IgG and IgG2a in the glomeruli. However, further TLR7/9 challenge has limited effects on IgG/IgG2a deposition. Minimal histopathological differences were observed among groups (Supplemental Table 1).

### EE exposure on serum autoantibodies and cytokine levels

**Figure 4:**
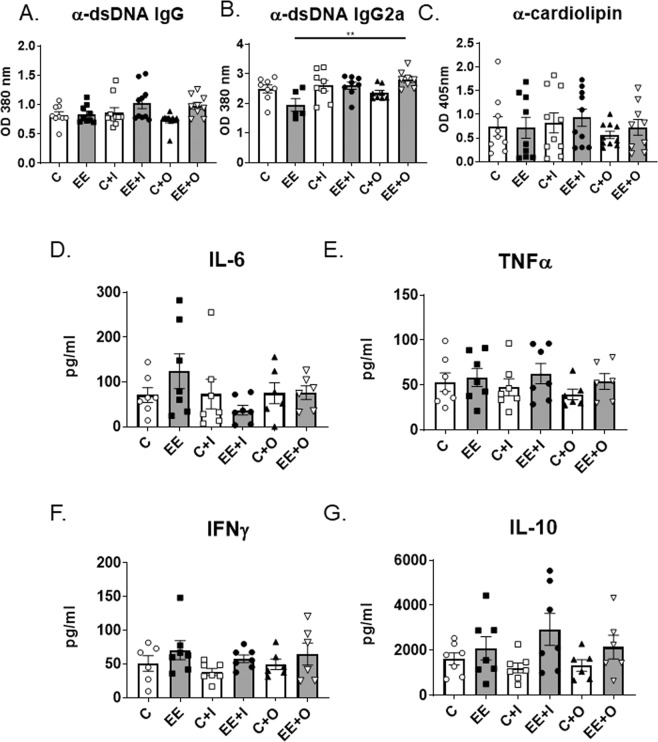
Serologic parameters of autoimmune disease. (**A–C**) Autoantibodies (**A**) anti-dsDNA IgG, (**B**) anti-dsDNA subset IgG2a and (**C**) anti-cardiolipin were analyzed by ELISA in serum of mice at 16 weeks of age. (**D–G**) Serum cytokines **(D**) IL-6, (E) TNFα, (**F**) IFNγ, and (**G**) IL-10 were measured by multiplex ELISA from Aushon at 16 weeks of age. Graph data are expressed as mean ± SEM (n = 9–10 per group). **p < 0.01.

We next evaluated circulating autoantibodies (anti-dsDNA and anti-cardiolipin) and cytokines in sera of experimental groups. Surprisingly, EE did not increase the levels of anti-dsDNA IgG compared to mice fed on control diet (Fig. 4A) (post-hoc power = 0.764). Serial kinetics data revealed minimal differences in total IgG anti-dsDNA among all groups throughout the study (Supplemental Fig. 1). Administration of secondary TLR9 agonist ODN 2395 however, led to significantly elevated circulating IgG2a anti-dsDNA levels compared to the EE-exposed mice without a secondary stimulation (Fig. 4B, EE vs EE + O). TLR7 agonist imiquimod administration to EE exposed mice resulted in a tendency for increased anti-dsDNA autoantibodies (but this was not statistically significant, EE *vs* EE + I). No differences were observed on either IgG or IgG2a anti-dsDNA antibodies among the mice groups exposed to control diet (Fig. 4A,B, C vs C + I vs C + O). No differences were found among all groups for circulating anti-cardiolipin (Fig. 4C) (post-hoc power = 0.161). These data reveal that EE markedly enhanced deposition of autoantibodies in the kidneys with no apparent changes in the circulating autoantibodies.

Levels of circulating cytokines can be an indication of a systemic inflammatory response. Interestingly, no differences were observed in the circulating levels of cytokines considered either pro-inflammatory (IFNγ [post-hoc power = 0.351], IL-6 [post-hoc power = 0.381], tumor necrosis factor (TNF)α [post-hoc power = 0.222]) or anti-inflammatory (IL-10) (post-hoc power = 0.588) among the various groups (Fig. 4D–G). When measuring IFNα in the serum, all samples were below detectable limits. These data suggest that although EE exposure appears to modulate the disease severity in a specific target organ, such as the kidney, this level of EE exposure does not promote a broad sustained systemic inflammatory response.

### EE exposure results in altered splenic TLR 7/9 expression

Secondary lymphoid organs are important sources to investigate autoimmune and inflammatory abnormalities in immune cells. Flow cytometry was performed on splenic leukocytes to evaluate T and B cell subsets, IFNγ receptor 1, and BAFF receptor cellular expression. No differences were identified among groups (data not shown).

**Figure 5:**
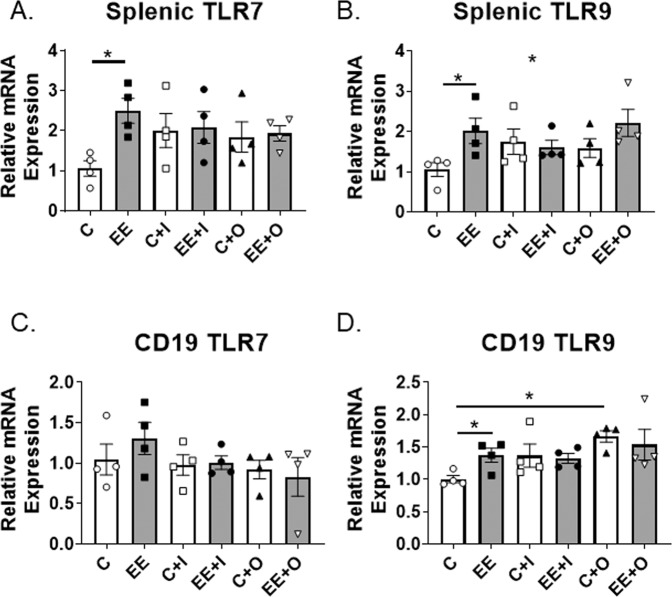
EE exposure can increase TLR7/9 expression in splenic leukocytes. (**A–D**) TLR7 and TLR9 mRNA expression levels were evaluated using qRT-PCR analysis of RNA collected from (**A,B**) splenic leukocytes and (**C,D**) splenic CD19^+^ cells. Expression levels are shown relative to the control group (labeled **C**) using comparative −2^ΔΔCt^. β-Actin was used at the endogenous control gene (n = 4 samples per group) Graph data are expressed as mean ± SEM, *p < 0.05.

Given that susceptibility to infections is one of the primary causes of morbidity and mortality in lupus patients, we evaluated splenic leukocytes to assess their ability to respond to viral and bacterial infections by using TLR7 and TLR9 agonists *in vivo* as a model of sterile inflammation. Mice fed the EE diet had increased mRNA expression of TLR7 and TLR9 in splenic leukocytes compared to mice fed the control diet (Fig. 5A,B, C vs EE). CD19^+^ cells had similar TLR7 expression (Fig. 5C, C vs EE) but increased TLR9 expression (Fig. 5D, C vs EE) in mice exposed to EE, when compared to the control group. TLR9 agonist increased TLR9 relative expression in CD19^+^ B cells only in mice fed the control diet, but not in EE-fed mice (Fig. 5D, C vs C + O, EE vs EE + O).

### EE exposure alters splenic cytokine production in response to *in vitro* TLR7/TLR9 stimulation

**Figure 6:**
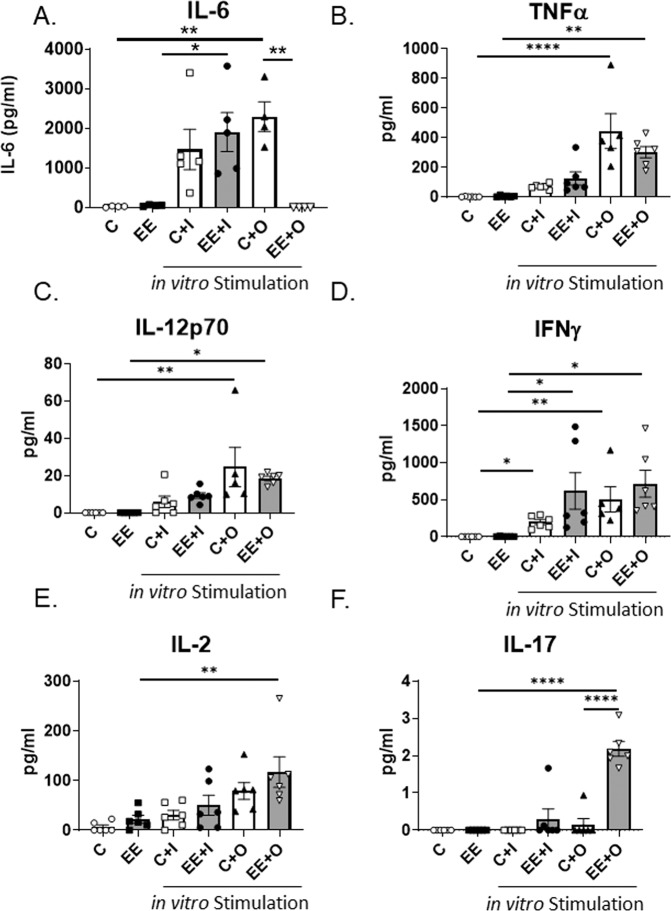
EE exposure modulates cytokine production. (**A–F**) Splenic leukocytes from Control diet and EE diet fed mice given *in vivo* sterile saline i.p. were activated *in vitro* with either imiquimod at 5 µg ml^−1^ or ODN 2395 (0.5 µM) for 24 hours. Cytokine protein levels were analyzed by ELISA from the cell culture supernatants. One-way ANOVA. Graph data are expressed as mean ± SEM (n = 6 per group). *p < 0.05, **p < 0.01, ****p < 0.0001.

We next evaluated the production of multiple pro-inflammatory cytokines commonly associated with SLE from splenic leukocytes following *in vitro* TLR7/9 stimulation. IL-6 protein production was suppressed in splenic leukocytes from mice exposed to EE and stimulated with TLR9 agonist *in vitro* (Fig. 6A, C + O vs EE + O). However, TLR7 imiquimod was able to induce IL-6 production in EE-exposed mice (Fig. 6A, C + I vs EE + I). This observation suggests that EE promotes differential TLR7 and TLR9 responses with regards to the induction of IL-6 (Fig. 6A). Both TLR7 and TLR9 agonists were able to elicit responses for TNFα, IL12-p70, IFNγ, IL-2, and IL-17 in both control and EE diet group, although some changes did not reach statistical significance. It was notable that in EE-fed mice, compared to TLR7 stimulation, TLR9 stimulation induced more prominent secretion levels of IL-12p70, IL-2, and IL-17 cytokines in supernatants (Fig. 6C,E,F). When measuring IFNα in the cell culture supernatants, all samples were below detectable limits. These results suggest that even though TLR9 expression can be increased in splenic leukocytes due to EE exposure or TLR9 stimulation, EE exposure can suppress certain TLR9 stimulated cytokine expression (such as IL-6).

Since EE exposure modulated *in vitro* TLR7 and/or TLR9 stimulated cytokine production in splenic leukocytes, we next assessed the TLR7 and TLR9 signaling cascade proteins in splenic leukocytes of C and EE diet exposed mice following *in vivo* TLR7 or TLR9 stimulation. Minimal differences were observed in splenic mRNA expression profiles following TLR7 and TLR9 stimulation *in vivo*. mRNA expression for Interleukin-1 receptor-associated kinase 4 (IRAK4), interferon regulatory factor 7 (IRF7), and TNF Receptor associated factor 6 (TRAF6) were evaluated in both splenic leukocytes and splenic CD19^+^ cells (Supplemental Fig. 2A–F). Due to differences in mRNA expression and protein production, we next evaluated signaling cascade protein levels by western blot analysis.

The protein levels of major signaling cascade proteins involved in TLR7/9 signaling were evaluated in splenic leukocyte whole cell lysates. Levels of IRAK4, phospho-IRAK4, TRAF6, and IRF7 were normalized to β-actin levels, an endogenous loading control. We also evaluated p65 levels in splenic leukocyte nuclear extracts, and normalized to proliferating cell nuclear antigen (PCNA). No significant differences were observed in protein levels of the signaling cascade proteins evaluated (Supplemental Fig. 3).

## Discussion

There is now extensive, indisputable evidence that the immune system is a target for estrogens^10,37–42^. Inconsistent with the high prevalence of indications for treatment with EE, there is a severe deficiency of published literature regarding EE’s effects on immune system modulation. This lack of investigations in EE-induced immunomodulation is especially obvious when considering the breadth of knowledge available for immunoregulatory effects of E2 and BPA. It is likely that individual response to vaccinations, infections and external antigenic stimuli can be influenced by the presence of endogenous as well as exogenous estrogenic compounds. In 2012 and 2013, the World Health Organization (WHO) published reports which concluded that evaluation of human exposure to EDCs and hormonally active pharmaceutical compounds must be addressed^43^. Concerns of EE exposure have been somewhat debatable following a Nurse’s Cohort study that described minimal differences seen with lupus patients and oral contraceptive use, while more recent studies have associated OCP use with increased SLE risk^44–46^. While EE is a major component of oral contraceptive pills, these medications also typically include a progesterone component as well. Depending on the context and cellular micro-environment, progesterone is generally considered to be anti-inflammatory or immune-suppressive, and may aid in masking or blocking the potentially harmful immunologic effects of EE^47,48^.

Two of the primary causes of mortality in SLE are lupus nephritis and infections. Our previous studies, in which EE was administered via silastic subcutaneous implants, supports a role for estrogenic chemicals in promotion of renal disease, and a role for EE in the suppression of TLR9 cytokine production^9^. We hypothesized that a subset of the population that is genetically prone to develop autoimmune diseases might be sensitive to the chronic environmental exposure to EE.

Exposure to exogenous estrogens likely occurs most commonly through oral ingestion. In this study, we developed a relevant model of oral EE exposure that involves the use of a grain-free purified-ingredients diet supplemented with 6.8 ppb of EE, a dose corresponding to a daily human exposure dose of 30 µg of EE. This eliminates unintended exposure to phytoestrogens and other common EEDC contaminants found in grain-based diet formulations^49^. This model also reduces mouse handling and the risks associated with daily oral gavage of chemicals of interest, while retaining the exposure of oral mucosa and microbiota to the EEDC of interest.

Remarkably, just a low-dose oral EE exposure of MRL/lpr mice accelerated and sustained renal disease as evidenced by increased proteinuria and BUN when compared to control diet fed mice (Figs. 1, 2). EE-fed mice had increased levels of proteinuria at the first time point studied (7 weeks of age) compared to control diet-fed mice (Fig. 1). This finding is notable considering that it is unusual for MRL/lpr mice to have high levels of proteinuria by 7 weeks of age. It is possible that EE-fed mice may have increased proteinuria even earlier than 7 weeks of age, a time point that was not studied. EE-fed mice had increased IgG and IgG2a glomerular immune complex deposition compared to controls (Fig. 3). Histopathology scores do not reflect the observed differences between groups in clinical markers of renal disease (Supplemental Table 1)^50^.

In our previous study, the estrogen-free diet formulation used in this study reduced histopathologic changes in kidneys of MRL/lpr mice^49^. It is possible that by using this dietary formulation the severe renal disease in MRL/lpr mice will be delayed. In this study, we found that a low oral dose of EE, remarkably, enhanced the deposition of autoantibodies in the kidneys. Even though histopathological changes in the kidneys were not evident at 16 weeks of age, it is also possible that these mice may develop renal histopathological changes later, since it is evident that immune complex deposition usually initiates and precedes anatomic alterations in renal tissue^19^. Ultra-structural changes may be occurring in the renal tissue that require a more advanced form of imaging, such as electron microscopy, to detect. This particular strain also suffers from severe lymphadenopathy and dermatitis which lead to wasting or animal welfare concerns, preventing the mice from aging beyond 16 weeks of age. Regardless, early clinical pathological (proteinuria, BUN, and BUN:Creatinine ratio) and immunofluorescent (immune complex deposition in the glomeruli) renal parameters may be an early prelude to histopathological changes.

Estrogens have previously been shown to promote B cell survival and break tolerance to produce autoantibodies in wild-type mice^6,41^. The exact mechanism of how estrogens lead to loss of B cell tolerance is still not fully understood. In this study, we did not observe any differences between groups in circulating cytokines (Fig. 4D–G), including BAFF or BAFF production following *in vitro* stimulation (Data not shown). Chronic exposure to EE may promote the survival of CD19^+^ B cells or plasma cells, leading to increased potential for autoantibody production. ODN 2395 is a form of double-stranded DNA, and the increased level of anti-dsDNA IgG2a autoantibodies was observed in mice exposed to EE and stimulated with ODN 2395 (Fig. 4B). This increase in anti-dsDNA IgG2a could be due to EE promoting B cell survival, followed by increased levels of dsDNA circulating throughout the body after administration of ODNs, leading to increased anti-dsDNA production. It is plausible that autoantibodies may be redirected from the serum and secondary lymphoid tissues to the target organ (kidney) hence, why we did not find markedly high levels of serum autoantibodies. It has been previously shown that during end-stage disease in mouse models of SLE, serum autoantibodies may actually decrease, relative to their peak concentration^51^. This also suggests that serum antibody levels alone may not be a good indicator of an individual’s disease status, and that all clinical parameters should be evaluated together to provide a clearer overall clinical assessment.

We cannot eliminate the possibility that EE promoted local target organ (kidney) *in situ* production of autoantibodies. Previous studies have shown the importance of infiltrating and extrafollicular B cell activation. Previously identified autoreactive DN2 B cells can be hyper-responsive and predominate in African American SLE patients with active disease and nephritis, and are plasma cell precursors^52^. Likely, these infiltrating autoantibody-producing cells are generated within the kidney tissue independent of formed germinal centers^52–54^. This suggests that renal immune complex deposition levels, as well as other target organ autoantibody deposition, may be independent of circulating autoantibody levels, requiring tissue biopsies to detect accurately.

The observed increased autoantibody production can be independent of TLR7/9 signaling, since autoantibody production does not require intact TLR7/9 signaling. Human SLE patients have impaired TLR9 response on PBMCs when evaluated *in vitro*^55,56^. We observed a similar suppression of TLR9 dependent IL-6 production in MRL/lpr, as we did in NZB/W_F1_ mice exposed to EE^9^. We did observe an increase in splenic leukocyte IL-2 and IL-17 production from EE exposed mice stimulated with TLR9 agonist (Fig. 6E,F). These changes could promote exacerbation of lupus disease and increase susceptibility to infections if the immune system cannot appropriately respond to pathogenic CpG DNA. IFNα is an important product following TLR7/9 stimulation, and the mouse model (MRL/lpr) used in this study, unfortunately, did not produce detectable levels of IFNα for further evaluation. It is important to consider that the autoimmune disease phenotype in MRL/lpr mice is mediated by IFNγ, but not IFNα/β.^57^ Previous work also suggests that EE exposure can increase TLR4 response to LPS stimulation *in vitro*^9^. Thus, mice exposed to EE that had gram negative bacteria as part of their normal microbiota could potentially have a heightened response to released LPS through TLR4 signaling compared to mice fed the control diet.

A limitation of this study is that we did not perform a formal sample size determination at the planning stage for any of the comparisons made. As such, non-significant findings should be interpreted with caution. For experiments with no significant findings, post-hoc overall power estimates are provided as a guide.

In summary, we investigated the effects of chronic exposure to a human-relevant dose of 17α-ethinyl estradiol on kidney disease and response to ssRNA viral (TLR7) or bacterial (TLR9) challenge in a mouse model of systemic lupus erythematosus. Chronic low-dose oral exposure to EE beginning in the pre-disease stage can exacerbate clinical markers of renal disease and promote glomerular IgG and IgG2a immune complex deposition. EE exposure led to altered cytokine production in splenic leukocytes. This study validated our recent studies that demonstrated feeding MRL/lpr mice a customized diet totally free of estrogens reduced immune complex renal disease. Here we show that deliberate addition of EE to this diet accelerated proteinuria production and exacerbated the immune complex deposition in the kidney. These data provide a significant step forward in our understanding of how environmental exposure to an estrogenic EDC can alter the immune system’s ability to respond to infectious organisms and modulate an already autoimmune-dysregulated immune system, exacerbating clinical evidence of renal disease even at an extremely low dose.

## Materials and Methods

### Mice and *in vivo* administration of TLR ligands

Genetically lupus-prone MRL/lpr (MRL/MpJ-*Fas*^*lpr*^/J, stock# 000485) mice were purchased from a single vendor, Jackson Laboratory (ME, USA). All mice were bred and housed in the AAALAC certified animal facility at the Virginia-Maryland College of Veterinary Medicine (VMCVM), Virginia Tech. Breeder mice were fed diet D11112226 (Research Diets Inc., New Brunswick, NJ, USA) to minimize the trans-generational effects of EEDC exposure from dietary sources. Diet D11112226, a purified-ingredients diet devoid of estrogenic chemicals was selected based on macronutrient sources. The protein source includes casein and L-Cystine, carbohydrates are from corn starch, maltodextrin 10, and dextrose, fiber is from Cellulose BW200 and inulin, fats are from soybean oil, and vitamins and minerals are from defined mixes. Due to the female predominance of lupus disease and increased exposure risk of females to EE in the form of prescription medications, for this study, we investigated the effects of chronic low-dose oral exposure to EE on female MRL/*lpr* mice only. At three (3) weeks of age, mice were weaned and randomly assigned to specific treatment groups to receive either D11112226 control diet (**C**, Research Diets Inc.) or the identically formulated custom diet, which incorporated 6.8 ppb of 17α-ethinyl estradiol into the D11112226 control diet (D16122101(B) or **EE**, Research Diets Inc.) This dose of EE was calculated based off of a human-relevant exposure dose (30 µg/day), and corrected for murine metabolic differences. This dose is within previously published doses of EE exposure in mice^58^. Calculations were corroborated with a board-certified toxicologist (Dr. M. Ehrich). Based on the results of our previous experiment, 10 mice were included in each treatment group to achieve sufficient power for statistical analysis to determine the autoimmune parameter change induced by EE diet (sixty total mice)^49^. Four mice did not reach the end of the study due to complications following blood collection or severe wounds from fighting. Data from these 4 mice were not included in the analysis. Mice were allowed to age and develop autoimmune disease until twelve (12) weeks of age. Mice were then administered an intraperitoneal injection of 100 uL of sterile saline containing 25 μg Imiquimod (TLR7 ligand, C + I and EE + I) every 48 hours, 5 μg ODN2395 (TLR9 ligand, C + O and EE + O) every 24 hours as previously reported^34,35^, or sterile saline alone (C and EE) as a control until 16 weeks of age. Mice were humanely euthanized at 16 weeks of age and tissues were collected. Throughout the study, mouse weights and diet consumption were measured weekly. Blood was collected by mandibular venipuncture and urine protein was evaluated every two weeks. Care was taken to ensure that environmental conditions for all groups were controlled, including mice being subjected to the same housing, local environment, temperature, ambient humidity, disinfectant used, bedding, and handling conditions. Paper-chip bedding was used to remove the risk of mycoestrogen exposure that can occur from corncob bedding materials^59^. Mice were housed with a light cycle of 12 hours of light and 12 hours of dark. All animal procedures and experiments were performed in accordance with guidelines of the Institutional Animal Care and Use Committee (IACUC) at Virginia Tech. The experimental protocols were approved by the Virginia Tech IACUC under protocol 17–191 CVM. Carbon dioxide asphyxiation followed by exsanguination was used for euthanasia as required by the approved IACUC protocol.

### Tissue preparation, and cellular culture

Whole splenic leukocytes were isolated using standard laboratory procedures described in detail previously^9,38,40,49,60,61^. Briefly, the spleens were dissociated by gently scraping through a steel screen, and the cell suspension was passed through a 70-μm cell strainer to remove tissue debris. To eliminate the unintended *in vitro* exposure to estrogens, care was taken to use only charcoal-stripped FBS and phenol red-deficient media. The splenic leukocytes were adjusted to 5×10^6^/ml in complete medium for seeding into cell culture plates for stimulation. Briefly, the cells were plated into 48-well cell culture plates (0.25 ml/well), and stimulated with Imiquimod (5 µg/ml) (IDT Inc., Skokie, IL, USA), ODN-2395 (0.5μM, synthesized by IDT Inc.), ODN Control (IDT Inc.), or complete media for the designated time by adding an equal volume of 2x concentration of stimulation medium. CD4^+^ and CD19^+^ cell were purified from whole splenic leukocytes using the Miltenyi Biotec “CD4^+^ (L3T4) MicroBeads, mouse,” and “CD19^+^ MicroBeads, mouse,” per the company’s published manual separation protocol (Miltenyi Biotec Inc., Auburn, CA, USA). Cell pellets were washed with cold PBS and stored at −80 °C.

### Measurement of proteinuria

Proteinuria was measured using Siemens Uristix dipsticks (Fisher, Hampton, NH, USA), or Chemistrip-2GP (Roche Diagnostics Corporation, Indianapolis, IN, USA). Urine was collected through manual restraint. The semi-quantitative scale was demonstrated as follows: “1”, 30 mg/dl; “2”, 100 mg/dl; and “3”, 300 mg/dl “4”, 500 mg/dL or over.

### Serum blood urea nitrogen and creatinine measurement

Blood was collected and allowed to clot for 30 minutes at room temperature. The blood samples were centrifuged at 2000xg for 10 minutes and serum was collected into new sterile 1.5 ml Eppendorf tubes. Serum was aliquoted and stored at −80 °C until used for assays. Blood urea nitrogen was measured using the commercial kit Urea Nitrogen (BUN) Colormetric Detection Kit (Thermofisher, Wilmington, DE, USA) as per the company’s published protocol. Serum creatinine was measured using the commercial kit Creatinine Assay Kit (Sigma, St. Louis, MO, USA) as per the included protocol. Two technical replicates were performed with each sample.

### Renal histopathology

The left kidneys from the MRL/lpr mice were collected and fixed in 10% buffered formalin and embedded in paraffin as previously described^49,51^. Five-micron sections were cut from paraffin embedded tissues, followed by staining with hematoxylin and eosin (H&E) or periodic acid-Schiff (PAS). This work was performed by AAVLD-accredited Virginia Tech Animal Laboratory Services (ViTALS) at Virginia-Maryland College of Veterinary Medicine, Virginia Tech. A board-certified pathologist assessed and scored the stained tissue slides in a blinded fashion. A grade of 0 to 4 (0 = perfect, no change; 1 = minimal; 2 = moderate; 3 = marked; and 4 = severe) was given to reflect the glomerular, tubular, interstitial, and vessel inflammation and lesions, respectively. The sum of the individual scores is reported as the total renal score, representing the microscopic changes in each sample^49,51^.

### Renal Immunofluorescence

The right kidneys were sectioned and one half of the cut kidney was immersed in Tissue-Tek O.C.T. Compound (Sakura Finetek, Torrance, CA, USA) and flash-frozen in a bath of dry ice and isopropanol. Frozen OCT samples were cut to 5 µm sections by ViTALS at Virginia-Maryland College of Veterinary Medicine and unstained slides were stored at −80 °C. Immunofluorescent slide preparation was performed as previously described^49^. The following anti-mouse antibodies were used in immunofluorescence analysis: complement C3-PE (RRID:AB_10557398, Cedarlane, Burlington, NC, USA); IgG-FITC (RRID:AB_465218, eBioscience, San Diego, CA, USA); IgG2a-Alexa Fluor 568 (RRID:AB_2535773), IgG3-Alexa Fluor 488 (RRID:AB_2535784, Thermo Fisher), anti-TLR7-PE (RRID:AB_2739295, BD Bioscience, San Jose, CA, USA), anti-TLR9-PE (RRID:AB_2739316, BD Bioscience). Kidney Sections were examined under a fluorescent microscope as previously described^49^. Fiji/ImageJ image processing program^62–64^ was used to trace the basement membranes and measure the fluorescent intensity of the selected area. The background fluorescence was then subtracted from the glomerular fluorescent intensity to determine the corrected glomerular fluorescent intensity (CGFI) value^49^. Twenty (20) glomeruli were evaluated per antibody, per sample.

### Assay of serum autoantibodies

#### Serum anti-dsDNA autoantibodies

The female MRL/lpr mice were aged in our facility and blood was collected by submandibular venipuncture every 2 weeks after they reached 7 weeks of age. The serum anti-dsDNA antibody levels were measured by ELISA per our previous reports^39,49,51,60^. The absorbance was measured by reading the plate at 380 nm with a SpectraMax M5 Microplate Reader (Molecular Devices, Sunnyvale, CA, USA). Two technical replicates were performed.

#### Serum anti-cardiolipin

End-point serum anti-cardiolipin levels were measured by ELISA as previously reported^49,51,60^. The absorbance was measured by reading the plate at 405 nm with a SpectraMax M5 Microplate Reader (Molecular Devices). Two technical replicates were performed.

### Cytokine/Chemokine ELISAs

The levels of BAFF in media, imiquimod, or ODN 2395 stimulated cell culture supernatants were analyzed using the Mouse BAFF/BLyS/TNFSF13B Quantikine ELISA Kit (R&D Systems, Minneapolis, MN, USA). The levels of IFNα in serum and splenic leukocyte culture supernatants were determined with eBioscience Mouse IFN alpha Platinum ELISA kit (eBioscience). The ELISAs were performed per the manufacturers’ protocols. Ciraplex® Chemiluminescent Assay kits (Aushon Biosystem, Billerica, MA, USA) were used to quantify the levels of IFN-γ, IL-1β, IL-2, IL-6, IL-10, and TNFα in serum and cell culture supernatants per the manufacturer’s instructions^49,65^. The images of chemiluminescent array plates were captured with Cirascan image system (Aushon) and the image data was processed with Cirasoft software. All the assays were performed with two technical replicates for each sample.

### RNA Extraction and qRT-PCR analysis of mRNA expression

Total RNA, containing small RNA, was isolated from whole splenic leukocytes and splenic CD19^+^ cells using a miRNeasy Mini Kit (Qiagen, Valencia, CA, USA) as described in our previous publications^9,49,51,65^. The RNA concentration was quantified using a NanoDrop 2000 (Thermo Fisher). As we described in detail previously^40,49,51,60^, Taqman Gene Expression Assay reagents (Applied Biosystems, Grand Island, NY, USA) were used to evaluate the mRNA expression levels of TRAF6, IRAK4, IRF7, TLR7, TLR9, and β-actin with two technical replicates for each sample. The expression levels of mRNAs were normalized to endogenous β-actin. The data was shown as relative expression level to an appropriate control by using the 2^−ΔΔCt^ formula (Livak method).

### Statistical analysis

All values in the graphs are given as mean ± SEM, or as otherwise stated in the figure legend. For proteinuria (the primary outcome), effects of diet, time, and the interaction between diet and time were assessed using mixed-model repeated measures ANOVA. The interaction between diet and time was further analyzed to assess the effects to diet at each time point. To assess statistical significance for the other outcomes, one-way ANOVA following by Tukey’s post-hoc method was performed for the group comparisons, unless otherwise specified in the figure legend. Statistical significance was set to p < 0.05. Statistical analyses were performed using SAS version 9.4 (Cary, NC, USA) and Prism Graphpad software (Version 8.0.2, La Jolla, CA, USA).

## Supplementary information

Supplemental Information.

## Data Availability

The datasets used and/or analyzed during the current study are available from the corresponding author on reasonable request.

## References

[1] Sciences, N. I. O. E. H. *Endocrine Disruptors*, https://www.niehs.nih.gov/health/topics/agents/endocrine/index.cfm (2019).

[2] DrugBank. *Ethinylestradiol*, https://www.drugbank.ca/drugs/DB00977 (2005).

[3] Caldwell DJ (2010). An assessment of potential exposure and risk from estrogens in drinking water. Environ Health Perspect.

[4] Lang TJ (2004). Estrogen as an immunomodulator. Clinical immunology.

[5] Ahmed SA (1999). Gender and risk of autoimmune diseases: possible role of estrogenic compounds. Environ Health Perspect.

[6] Ahmed SA (1989). Estrogen induces the development of autoantibodies and promotes salivary gland lymphoid infiltrates in normal mice. Journal of autoimmunity.

[7] Ahmed SA (2000). The immune system as a potential target for environmental estrogens (endocrine disrupters): a new emerging field. Toxicology.

[8] Ahmed SA, Verthelyi D (1993). Antibodies to cardiolipin in normal C57BL/6J mice: induction by estrogen but not dihydrotestosterone. Journal of autoimmunity.

[9] Dai R, Edwards MR, Heid B, Ahmed SA (2019). 17beta-Estradiol and 17alpha-Ethinyl Estradiol Exhibit Immunologic and Epigenetic Regulatory Effects in NZB/WF1 Female Mice. Endocrinology.

[10] Dai R (2017). Neutrophils and neutrophil serine proteases are increased in the spleens of estrogen-treated C57BL/6 mice and several strains of spontaneous lupus-prone mice. PloS one.

[11] Dragin N (2016). Estrogen-mediated downregulation of AIRE influences sexual dimorphism in autoimmune diseases. The Journal of clinical investigation.

[12] Dragin N (2017). Balance between Estrogens and Proinflammatory Cytokines Regulates Chemokine Production Involved in Thymic Germinal Center Formation. Scientific reports.

[13] Panchanathan R, Liu H, Leung YK, Ho SM, Choubey D (2015). Bisphenol A (BPA) stimulates the interferon signaling and activates the inflammasome activity in myeloid cells. Mol Cell Endocrinol.

[14] Kharrazian D (2014). The Potential Roles of Bisphenol A (BPA) Pathogenesis in Autoimmunity. Autoimmune Dis.

[15] Dai, R., Edwards, M. R., Heid, B. & Ahmed, S. A. Data from: 17-β estradiol and synthetic 17α-ethinyl estradiol exhibit distinct immunologic and epigenetic regulatory effects in NZB/WF1 mice. *VTechData Repository*, pending (2018).

[16] Union, E. Vol. L 226 56 [E.Pa.C.o.t.E. Union (ed.)] 1-17 (Off. J. Eur. Union, 2013).

[17] Ward MM, Pyun E, Studenski S (1995). Causes of death in systemic lupus erythematosus. Long-term followup of an inception cohort. Arthritis and rheumatism.

[18] Wang Z (2015). Long-term survival and death causes of systemic lupus erythematosus in China: a systemic review of observational studies. Medicine (Baltimore).

[19] Wallace, D. J., Hahn, B. & Dubois, E. L. *Dubois’ lupus erythematosus and related syndromes*. 8th edn, (Elsevier/Saunders, 2013).

[20] James Judith A., Neas Barbara R., Moser Kathy L., Hall Teresa, Bruner Gail R., Sestak Andrea L., Harley John B. (2001). Systemic lupus erythematosus in adults is associated with previous Epstein-Barr virus exposure. Arthritis & Rheumatism.

[21] James JA, Robertson JM (2012). Lupus and Epstein-Barr. Current opinion in rheumatology.

[22] Lossius A, Johansen JN, Torkildsen O, Vartdal F, Holmoy T (2012). Epstein-Barr virus in systemic lupus erythematosus, rheumatoid arthritis and multiple sclerosis-association and causation. Viruses.

[23] Nickerson KM (2010). TLR9 regulates TLR7- and MyD88-dependent autoantibody production and disease in a murine model of lupus. Journal of immunology.

[24] Koh YT (2013). Role of nucleic acid-sensing TLRs in diverse autoantibody specificities and anti-nuclear antibody-producing B cells. Journal of immunology.

[25] Santiago-Raber ML (2010). Critical role of TLR7 in the acceleration of systemic lupus erythematosus in TLR9-deficient mice. Journal of autoimmunity.

[26] Summers SA (2010). TLR9 and TLR4 are required for the development of autoimmunity and lupus nephritis in pristane nephropathy. Journal of autoimmunity.

[27] Desnues B (2014). TLR8 on dendritic cells and TLR9 on B cells restrain TLR7-mediated spontaneous autoimmunity in C57BL/6 mice. Proceedings of the National Academy of Sciences of the United States of America.

[28] Andrews BS (1978). Spontaneous murine lupus-like syndromes. Clinical and immunopathological manifestations in several strains. The Journal of experimental medicine.

[29] Hang L, Theofilopoulos AN, Dixon FJ (1982). A spontaneous rheumatoid arthritis-like disease in MRL/l mice. The Journal of experimental medicine.

[30] Theofilopoulos AN (1980). A comparative immunologic analysis of several murine strains with autoimmune manifestations. Clin Immunol Immunopathol.

[31] Theofilopoulos AN, Dixon FJ (1985). Murine models of systemic lupus erythematosus. Adv Immunol.

[32] Kelley VE, Roths JB (1985). Interaction of mutant lpr gene with background strain influences renal disease. Clin Immunol Immunopathol.

[33] Cohen PL, Eisenberg RA (1991). Lpr and gld: single gene models of systemic autoimmunity and lymphoproliferative disease. Annual review of immunology.

[34] Pawar RD (2006). Toll-like receptor-7 modulates immune complex glomerulonephritis. Journal of the American Society of Nephrology: JASN.

[35] Longhini AL (2014). *In vivo* administration of TLR9 agonist reduces the severity of experimental autoimmune encephalomyelitis. The role of plasmacytoid dendritic cells and B lymphocytes. CNS Neurosci Ther.

[36] Anders HJ (2003). Bacterial CpG-DNA aggravates immune complex glomerulonephritis: role of TLR9-mediated expression of chemokines and chemokine receptors. Journal of the American Society of Nephrology: JASN.

[37] Khan D, Ansar Ahmed S (2016). The Immune System Is a Natural Target for Estrogen Action: Opposing Effects of Estrogen in Two Prototypical Autoimmune Diseases. Frontiers in immunology.

[38] Dai R, Phillips RA, Karpuzoglu E, Khan D, Ahmed SA (2009). Estrogen regulates transcription factors STAT-1 and NF-kappaB to promote inducible nitric oxide synthase and inflammatory responses. Journal of immunology.

[39] Khan D, Dai R, Karpuzoglu E, Ahmed SA (2010). Estrogen increases, whereas IL-27 and IFN-gamma decrease, splenocyte IL-17 production in WT mice. European journal of immunology.

[40] Dai R (2008). Suppression of LPS-induced Interferon-gamma and nitric oxide in splenic lymphocytes by select estrogen-regulated microRNAs: a novel mechanism of immune modulation. Blood.

[41] Bynoe MS, Grimaldi CM, Diamond B (2000). Estrogen up-regulates Bcl-2 and blocks tolerance induction of naive B cells. Proceedings of the National Academy of Sciences of the United States of America.

[42] Penhale WJ, Ahmed SA (1982). Animal model of human disease. Lymphocytic thyroiditis. Autoimmune thyroiditis in rats induced by thymectomy and irradiation. The American journal of pathology.

[43] Programme, W. W. H. O. U. U. N. E. *The-State-of-the-Science of endocrine disrupting chemicals*, http://www.who.int/ceh/publications/endocrine/en/index.html (2013).

[44] Sanchez-Guerrero, J. *et al*. Past use of oral contraceptives and the risk of developing systemic lupus erythematosus. *Arthritis and rheumatism* **40**, 804–808, 10.1002/1529-0131(199705)40:5&lt;804::AID-ART4&gt;3.0.CO;2-E (1997). 10.1002/art.1780400505 9153539

[45] Bernier MO, Mikaeloff Y, Hudson M, Suissa S (2009). Combined oral contraceptive use and the risk of systemic lupus erythematosus. Arthritis and rheumatism.

[46] Costenbader KH, Feskanich D, Stampfer MJ, Karlson EW (2007). Reproductive and menopausal factors and risk of systemic lupus erythematosus in women. Arthritis and rheumatism.

[47] Hughes GC (2012). Progesterone and autoimmune disease. Autoimmunity reviews.

[48] Thongngarm T, Jenkins JK, Ndebele K, McMurray RW (2003). Estrogen and progesterone modulate monocyte cell cycle progression and apoptosis. Am J Reprod Immunol.

[49] Edwards MR (2017). Commercial rodent diets differentially regulate autoimmune glomerulonephritis, epigenetics and microbiota in MRL/lpr mice. Int Immunol.

[50] Edwards, M. *et al*. Data From: Low-dose EE exposure exacerbates lupus renal disease and modulates tissue-specific responses to TLR7/9 agonists in genetically autoimmune-prone mice. *VTechData Repository*, Pending (2019). 10.1038/s41598-020-62124-6 PMC7090002 32251357

[51] Dai R (2013). Sex differences in the expression of lupus-associated miRNAs in splenocytes from lupus-prone NZB/WF1 mice. Biology of sex differences.

[52] Jenks SA (2018). Distinct Effector B Cells Induced by Unregulated Toll-like Receptor 7 Contribute to Pathogenic Responses in Systemic Lupus Erythematosus. Immunity.

[53] Jenks SA, Cashman KS, Woodruff MC, Lee FE, Sanz I (2019). Extrafollicular responses in humans and SLE. Immunol Rev.

[54] Wang S (2018). IL-21 drives expansion and plasma cell differentiation of autoreactive CD11c(hi)T-bet(+) B cells in SLE. Nat Commun.

[55] Mortezagholi S (2016). Evaluation of PBMC Distribution and TLR9 Expression in Patients with Systemic Lupus Erythematosus. Iran J Allergy Asthma Immunol.

[56] Mortezagholi S (2017). Evaluation of TLR9 expression on PBMCs and CpG ODN-TLR9 ligation on IFN-alpha production in SLE patients. Immunopharmacol Immunotoxicol.

[57] Balomenos D, Rumold R, Theofilopoulos AN (1998). Interferon-gamma is required for lupus-like disease and lymphoaccumulation in MRL-lpr mice. The Journal of clinical investigation.

[58] Gear RB, Belcher SM (2017). Impacts of Bisphenol A and Ethinyl Estradiol on Male and Female CD-1 Mouse Spleen. Scientific reports.

[59] Thigpen JE (2013). The estrogenic content of rodent diets, bedding, cages, and water bottles and its effect on bisphenol A studies. J Am Assoc Lab Anim Sci.

[60] Dai R (2010). Identification of a common lupus disease-associated microRNA expression pattern in three different murine models of lupus. PloS one.

[61] Dai R, Phillips RA, Ahmed SA (2007). Despite Inhibition of Nuclear Localization of NF- B p65, c-Rel, and RelB, 17- Estradiol Up-Regulates NF- B Signaling in Mouse Splenocytes: The Potential Role of Bcl-3. The Journal of Immunology.

[62] Schindelin J (2012). Fiji: an open-source platform for biological-image analysis. Nature methods.

[63] Schneider CA, Rasband WS, Eliceiri KW (2012). NIH Image to ImageJ: 25 years of image analysis. Nature methods.

[64] Schindelin J, Rueden CT, Hiner MC, Eliceiri KW (2015). The ImageJ ecosystem: An open platform for biomedical image analysis. Mol Reprod Dev.

[65] Dai R, Lu R, Ahmed SA (2016). The Upregulation of Genomic Imprinted DLK1-Dio3 miRNAs in Murine Lupus Is Associated with Global DNA Hypomethylation. PloS one.

