# Supplementary material for: Low-dose 17α-ethinyl estradiol (EE) exposure exacerbates lupus renal disease and modulates immune responses to TLR7/9 agonists in genetically autoimmune-prone mice
Source: Sci Rep. 2020 Mar 23;10:5210. doi: 10.1038/s41598-020-62124-6 (PMC7090002; doi:10.1038/s41598-020-62124-6)
Supplement: Supplementary file 1 — Supplemental Information. [file 41598_2020_62124_MOESM1_ESM.docx]

**Low-dose 17α-ethinyl estradiol (EE) exposure exacerbates lupus renal disease and modulates immune responses to TLR7/9 agonists in genetically autoimmune-prone mice** *Michael R. Edwards, Rujuan Dai, Bettina Heid, Catharine Cowan, Stephen R. Werre, Thomas Cecere, S. Ansar Ahmed^*^*

Supplemental Table I.

| **Group** | **Glomeruli** | **Tubules** | **Interstitium** | **Vessels** | **Total Scores** |
| --- | --- | --- | --- | --- | --- |
| Control Diet | 1.222±0.8333 | 0.222±0.441 | 0.333±0.500 | 2.222±0.441 | 4.000±1.581 |
| EE Diet | 1.222±0.667 | 0.667±0.866 | 0.778±1.093 | 2.111±0.333 | 4.778±2.728 |
| Control + Imiquimod | 2.000±0.943 | 0.800±1.033 | 0.900±0.994 | 2.300±0.483 | 6.000±3.055 |
| EE + Imiquimod | 1.200±0.789 | 0.400±0.516 | 0.600±0.516 | 2.200±0.422 | 4.400±1.955 |
| Control + ODN 2395 | 1.400±0.966 | 0.500±0.707 | 0.500±0.527 | 2.100±0.316 | 4.500±1.841 |
| EE + ODN 2395 | 1.667±0.866 | 0.889±1.054 | 0.667±0.707 | 2.222±0.441 | 5.444±2.833 |


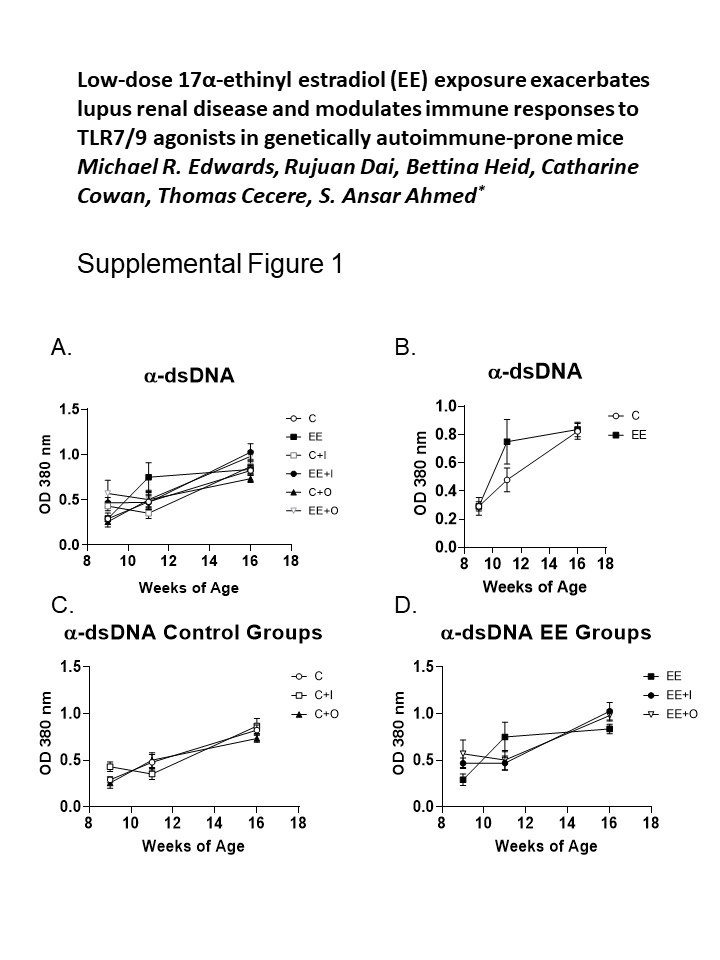


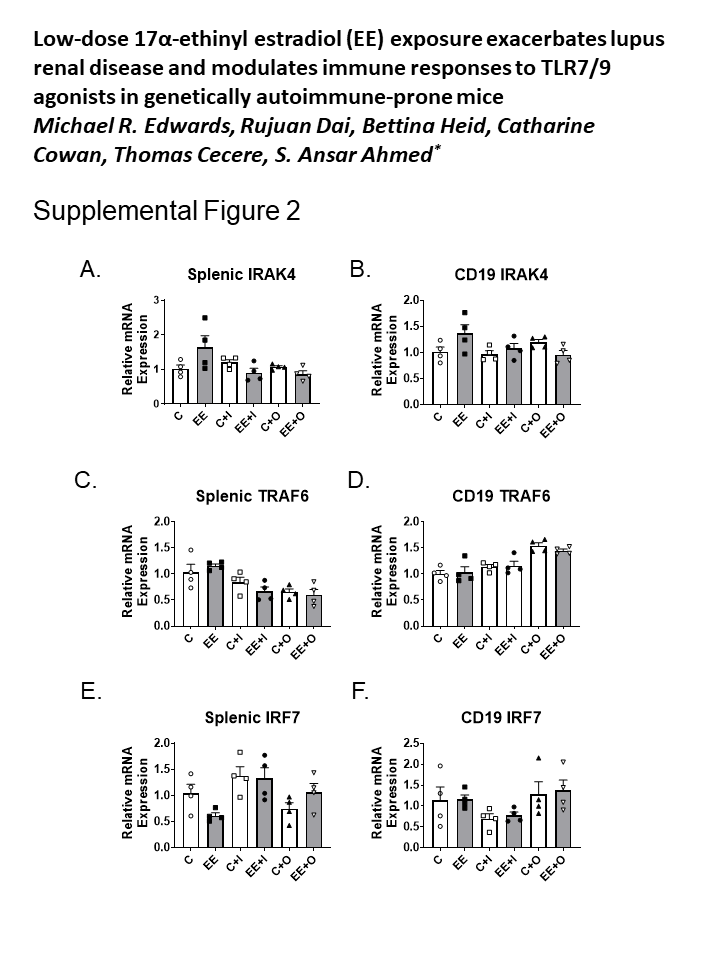


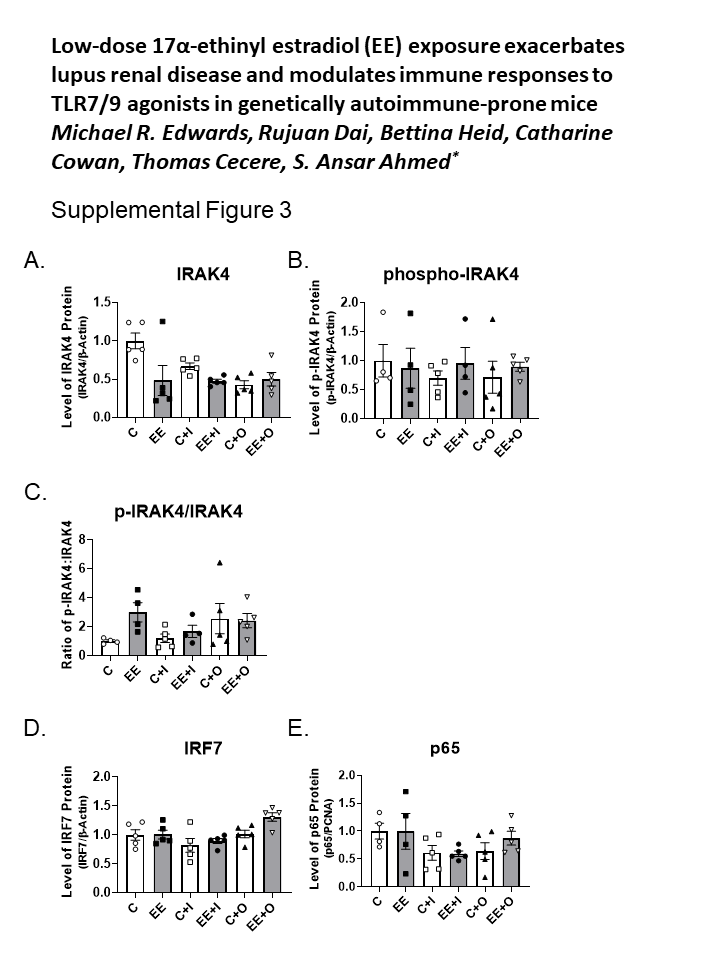


**Supplemental Figure Legends**

**Supplemental Figure 1**- Circulating anti-dsDNA throughout the study. Autoantibodies anti-dsDNA IgG was analyzed by ELISA in serum of mice at 9, 11, and 15 weeks of age. Data is shown for all treatment groups combined. Graph data are expressed as mean ± SEM (weeks 9, 11: n= 5-6, week 15: n=9-10 per group).

**Supplemental Figure 2**- mRNA expression of TLR7/9 signaling cascade proteins are similar among groups. (A-F) mRNA expression levels were evaluated using qRT-PCR analysis of RNA extracted from unstimulated splenic leukocytes. Expression levels are shown relative to the control group using comparative -2^ΔΔCt^. β-Actin was used at the endogenous control gene. One-way ANOVA. Graph data are expressed as mean ± SEM (n=5 per group).

**Supplemental Figure 3-** Signaling cascade protein levels are unchanged by EE exposure or *in vivo* TLR7/9 agonist administration. Protein was extracted from unstimulated splenic leukocytes and analyzed by western blot for IRAK4, p-IRAK4, IRF7, p65, and β-actin (loading control). (A) Densitometry evaluation of IRAK4, (B) p-IRAK4, (C) ratio of p-IRAK4 to IRAK4, and (D) IRF7 blot images with background subtracted prior to normalization to β-actin or (E) p65 normalized to PCNA, as analyzed through Fiji/ImageJ. Graph data are expressed as mean ± SEM (n=5 per group).

**Supplemental Table I Legend**- Scores of formalin-fixed kidney slides. H&E- and PAS-stained slides were analyzed by a board-certified veterinary pathologist in a blinded fashion. Data is represented as mean ± SD for each individual renal tissue structure. Total Kidney scores represents the sum of individual tissue sections scores. (n=9-10 mice per group).
